# Luteolin protects DYT-*PRKRA* cells from apoptosis by suppressing PKR activation

**DOI:** 10.3389/fphar.2023.1118725

**Published:** 2023-02-15

**Authors:** Kenneth Frederick, Rekha C. Patel

**Affiliations:** Department of Biological Sciences, University of South Carolina, Columbia, SC, United States

**Keywords:** dystonia, DYT16, DYT-PRKRA, PRKRA, eIF2α, PKR, PACT, ISR

## Abstract

DYT-*PRKRA* is a movement disorder caused by mutations in the *PRKRA* gene, which encodes for PACT, the protein activator of interferon-induced, double-stranded RNA (dsRNA)-activated protein kinase PKR. PACT brings about PKR’s catalytic activation by a direct binding in response to stress signals and activated PKR phosphorylates the translation initiation factor eIF2α. Phosphorylation of eIF2α is the central regulatory event that is part of the integrated stress response (ISR), an evolutionarily conserved intracellular signaling network essential for adapting to environmental stresses to maintain healthy cells. A dysregulation of either the level or the duration of eIF2α phosphorylation in response to stress signals causes the normally pro-survival ISR to become pro-apoptotic. Our research has established that the *PRKRA* mutations reported to cause DYT-*PRKRA* lead to enhanced PACT-PKR interactions causing a dysregulation of ISR and an increased sensitivity to apoptosis. We have previously identified luteolin, a plant flavonoid, as an inhibitor of the PACT-PKR interaction using high-throughput screening of chemical libraries. Our results presented in this study indicate that luteolin is markedly effective in disrupting the pathological PACT-PKR interactions to protect DYT-*PRKRA* cells against apoptosis, thus suggesting a therapeutic option for using luteolin to treat DYT-*PRKRA* and possibly other diseases resulting from enhanced PACT-PKR interactions.

## 1 Introduction

Dystonia is a diverse group of movement disorders that involve repetitive, often painful movements of affected body parts resulting in abnormal gaits and postures (Grütz and Klein, 2021). Several forms of inherited, monogenic dystonia have been characterized (Weisheit et al., 2018) and one such type is DYT-*PRKRA* (aka DYT16), caused by mutations in the *PRKRA* gene, which encodes the protein PACT (Patel and Sen, 1998). DYT-*PRKRA* is a rare, childhood-onset condition that exhibits progressive limb, laryngeal, and oromandibular dystonia with features of parkinsonism. Eleven mutations causing DYT-*PRKRA* have been identified thus far in the *PRKRA* gene (OMIM: DYT16, 612067) (Camargos et al., 2008; Seibler et al., 2008; Camargos et al., 2012; Lemmon et al., 2013; Zech et al., 2014; de Carvalho Aguiar et al., 2015; Quadri et al., 2016; Dos Santos et al., 2018; Masnada et al., 2021; Bhowmick et al., 2022). Although most *PRKRA* mutations causing dystonia are recessive, four dominantly inherited variants have also been reported so far (Seibler et al., 2008; Zech et al., 2014).

PACT is an activator of protein kinase PKR in response to a variety of stress signals that include endoplasmic reticulum (ER) stress, oxidative stress, osmolarity changes, and serum deprivation (Ito et al., 1999; Patel et al., 2000; Bennett et al., 2004; Bennett et al., 2012; Farabaugh et al., 2020). PKR is a double-stranded RNA (dsRNA)-activated protein kinase, which is ubiquitously expressed, and its expression is induced by antiviral cytokine interferon (IFN) (Meurs et al., 1990; Garcia et al., 2007). The kinase activity of PKR remains latent until it binds to an activator, which brings about a conformational change to expose the ATP-binding site and PKR’s enzymatic activation (Nanduri et al., 1998; Cole, 2007). In virus infected cells PKR is activated by direct interactions with dsRNA, a viral replication intermediate or virally encoded RNA with extensive ds structures (Barber, 2001). However, in uninfected cells, stress signals activate PKR *via* its protein activator, PACT (Patel and Sen, 1998) in a dsRNA-independent manner. Once activated, PKR phosphorylates the *α* subunit of the eukaryotic translation initiation factor 2 (eIF2α) on serine 51 resulting in a transient attenuation of general protein synthesis (Garcia et al., 2006) and this response is part of the integrated stress response (ISR) pathway. ISR is an evolutionarily conserved pathway activated in eukaryotic cells by diverse stress signals that functions mainly to restore cellular homeostasis and recovery from stress (Pakos-Zebrucka et al., 2016). One of the four serine/threonine kinases phosphorylate eIF2α and each one of these kinases responds to a specific stress signal sometimes acting in an overlapping manner (Donnelly et al., 2013; Taniuchi et al., 2016). Phosphorylation of eIF2α prevents the formation of the ternary complex required for translation initiation, leading to a significant decrease in general protein synthesis but at the same time promoting the selective translation of specific mRNAs encoding proteins that promote cellular recovery (Wek, 2018). Although transient eIF2α phosphorylation promotes cellular survival, prolonged eIF2α phosphorylation induces apoptosis due to the transcriptional induction as well as preferential translation of pro-apoptotic transcripts (Donnelly et al., 2013). Thus, the pro-survival ISR response can become pro-apoptotic after exposure to severe or chronic stress to regulate the cellular stress response depending on the duration or severity of the initiating stress signal.

Previously, our lab reported that four recessively inherited and two dominantly inherited PACT substitution mutations increase cell susceptibility to ER stress by causing elevated levels of PKR activation and eIF2α phosphorylation that also persist for a longer duration in DYT-*PRKRA* patient-derived lymphoblasts (Vaughn et al., 2015; Burnett et al., 2020). Furthermore, a truncated PACT protein resulting from a dominantly inherited frameshift mutation, increased PACT-mediated PKR activation, and an enhanced sensitivity to ER stress also *via* causing PKR activation and eIF2α phosphorylation (Burnett et al., 2019). Based on these earlier studies, elevated activation of PKR emerged as a common theme for the PACT mutations reported to cause DYT-*PRKRA*, thus indicating that inhibition of PKR may be able to restore normal ISR and protect against increased apoptosis in dystonia patient cells. Several hyperactive PKR mutations were also reported recently to cause early-onset dystonia especially after a febrile illness (Kuipers et al., 2021; Musacchio et al., 2021; Magrinelli et al., 2022; Waller et al., 2022). Based on our previous research on DYT-*PRKRA* and reports of abnormally high PKR activation in early onset dystonia, it is of interest to evaluate if inhibition of PKR can protect DYT-*PRKRA* cells from increased apoptosis. In this study, we have used tunicamycin to induce ER stress and assess if PKR inhibition can protect the cells from apoptosis. A global inhibition of PKR by a chemical inhibitor could be detrimental in patients as PKR activation is an essential component of an innate antiviral response that is required to ward off severe consequences of viral infections (Hull and Bevilacqua, 2016; Hartmann, 2017; Cesaro and Michiels, 2021). Thus, a specific compound that could work by the disruption of PACT-PKR interaction may be best suited for clinical use. Our previous research has identified plant flavonoid luteolin as a compound that disrupts PACT-PKR interactions (Dabo et al., 2017; Burnett et al., 2020). Thus, we investigated the effect of luteolin on DYT-*PRKRA* cells after ER stress and our results indicate that luteolin protects DYT-*PRKRA* patient cells after ER stress by disruption of pathological PACT-PKR interactions while allowing stress-induced transient PACT-PKR interactions to restore the normal, protective ISR response.

## 2 Materials and methods

### 2.1 Cell lines, chemicals, and antibodies

Both HeLaM and COS-1 cells were cultured Dulbecco’s Modified Eagle’s Medium (DMEM) containing 10% Fetal Bovine Serum and penicillin/streptomycin. wt and DYT16 Patient B-Lymphoblasts were cultured in RPMI 1640 medium containing 10% FBS and penicillin/streptomycin. Both wt and DYT-*PRKRA* patient lymphoblast cell lines were Epstein-Barr Virus-transformed to create stable cell lines as previously described by Dr. Nutan Sharma (Mass Gen. Hospital), who kindly provided them to us (Anderson and Gusella, 1984; Vaughn et al., 2015). In this study we used the compound heterozygous DYT-*PRKRA* patient lymphoblasts that carried a P222L mutation as one allele and C213R mutation as a second allele. All transfections were carried using Effectene transfection reagent (Qiagen) per manufacturer protocol. The antibodies used were as follows: PKR: anti-PKR (human) monoclonal (71/10, R&D Systems), P-PKR: anti-phospho-PKR (Thr-446) monoclonal (Abcam [E120]), eIF2α: anti-eIF2α polyclonal (Invitrogen, AHO1182), p-eIF2α: anti-phospho-eIF2α (Ser-51) polyclonal (CST, #9721), PACT: Anti-PACT monoclonal (Abcam, ab75749), ATF4: Anti-ATF4 monoclonal (CST, #11815), CHOP: anti-CHOP monoclonal (CST, #2895), Cleaved PARP-1: anti-Cleaved-PARP monoclonal (CST, #32563), β-Actin: Anti- β-Actin-Peroxidase monoclonal (Sigma-Aldrich, A3854). Luteolin (sc-203119C) and tunicamycin (sc-203119C) was purchased from Santa Cruz Biotechnology. C16 was purchased from Sigma-Aldrich (527450).

### 2.2 PKR activity assays

HeLa M cells treated with IFN-β for 24-h and harvested at 70% confluency, washed using ice-cold PBS and centrifuged at 600 g for 5-min. Cell were resuspended in lysis buffer (20 mM Tris–HCl pH 7.5, 5 mM MgCl2, 50 mM KCl, 400 mM NaCl, 2 mM DTT, 1% Triton X-100, 100 U/ml aprotinin, 0.2 mM PMSF, 20% glycerol) and incubated on ice for 5 min. Lysates were centrifuged at 10,000 g for an additional 5-min. PKR was immunoprecipitated from 100 µg of this protein extract using anti-PKR monoclonal antibody (R&D Systems: MAB 1980) in a high salt buffer (20 mM Tris–HCl pH 7.5, 50 mM KCl, 400 mM NaCl, 1 mM EDTA, 1 mM DTT, 100 U/ml aprotinin, 0.2 mM PMSF, 20% glycerol, 1% Triton X-100) at 4°C on a rotating wheel for 30-min. We then added 10 µL of protein A-Sepharose beads to each immunoprecipitate followed by an additional 1 h incubation under the same conditions. Protein A-Sepharose beads were washed 4 times in high salt buffer followed by an additional two washes in activity buffer (20 mM Tris–HCl pH 7.5, 50 mM KCl, 2 mM MgCl2, 2 mM MnCl2, 100 U/ml aprotinin, 0.1 mM PMSF, 5%, glycerol). PKR activity assay using PKR bound to protein A-Sepharose beads was conducted by using 10 µL activity buffer containing 0.1 mM ATP and 10 µCi of [γ-^32^P] ATP. Either no activator, pure recombinant wt PACT (4 ng) or polyI:polyC dsRNA (400 pg) were used as the PKR activator and were added to the activity buffer befor the addition of ATP. Reaction was incubated at 30°C for 10 min and resolved on a 12% SDS-PAGE gel followed by phosphorimager analysis on Typhoon FLA7000.

### 2.3 Western blot analysis

Lymphoblasts derived from a compound heterozygous DYT16 patient containing both P222L and C213R mutations as independent alleles were cultured alongside lymphoblasts derived from a family member containing no mutations in PACT as our control wt cells. Cells were plated at a concentration of 300,000 cells/ml of RPMI media containing 10% fetal bovine serum and penicillin/streptomycin. To analyze cellular response to ER stress, we treated cells with 5 μg/ml of tunicamycin (Santa Cruz) over a 24-h time course and harvested cells in RIPA (150 mM NaCl, 1.0% IGEPAL^®^ CA-630, 0.5% sodium deoxycholate, 0.1% SDS, 50 mM Tris, pH 8.0) buffer containing a 1:100 dilution of protease inhibitor cocktail (Sigma) and phosphatase inhibitor (Sigma). Concentration of total protein extract was then determined using BCA assay and appropriate amounts of extracts were analyzed by western blot analyses using appropriate antibodies as indicated. When the cells were treated with luteolin prior to tunicamycin treatment, luteolin was added at 50 µM for 24 h. Quantification of band intensities was done using the Imagequant TL (Cytiva) software.

### 2.4 Co-Immunoprecipitation assays with endogenous proteins

For Co-Immunoprecipitation (co-IP) of endogenous proteins DYT-*PRKRA* and wt lymphoblasts were seeded at a concentration of 300,000 cells/ml of RPMI complete media and either left untreated or treated with 50 µM of luteolin (Santa Cruz) for 24 h. When treated with tunicamycin for indicated time periods after luteolin treatment, tunicamycin was added at 5 μg/ml. Cells were harvested at indicated time points and whole cell extract was immunoprecipitated overnight at 4°C on a rotating wheel in IP buffer (20 mM Tris-HCl pH 7.5, 150 mM NaCl, 1 mM EDTA, 1% Triton X-100, 20% Glycerol) using anti-PKR antibody (71/10, R&D Systems) and protein A sepharose beads (GE Healthcare). Immunoprecipitation was carried out using 100 ng of anti-PKR antibody and 10 µL of protein A sepharose beads slurry per immunoprecipitation. Immunoprecipitates were washed 3 times in 500 µL of IP buffer followed by resuspension and boiling for 5 min in 1X Laemmli buffer (150 mM Tris–HCl pH 6.8, 5% SDS, 5% β-mercaptoethanol, 20% glycerol). Samples were resolved on 10% SDS-PAGE denaturing gel and probed with anti-PACT antibody to determine co-IP efficiency and anti-PKR antibody to determine equal amounts of PKR were immunoprecipitated in each sample. Input blots of whole cell extract without immunoprecipitation are shown to indicate equal amounts of protein in each sample.

### 2.5 Mammalian 2-hybrid interaction assays

In all cases, wt PACT, P222L, C213R, DD (S246D, S287D) mutant PACT, or PKR ORFs were sub-cloned into both pSG424 expression vector such that it created an in-frame fusion to a GAL4 DNA binding domain (GAL4-DBD), and pVP16AASV19N expression vector such that it maintains an in-frame fusion to the activation domain of the herpes simplex virus protein VP16 (VP16-AD). All these plasmids have been described in our earlier publications (Vaughn et al., 2015; Burnett et al., 2020). COS-1 cells were then transfected with: i) 250 ng each of the GAL4-DBD and the VP16-AD constructs, ii) 50 ng of pG5LUC a firefly luciferase reporter construct, and iii) 1 ng of pRLNull plasmid (Promega), to normalize for transfection efficiencies. Cells were then harvested 24-h post transfection and assayed for both firefly and renilla luciferase activities using Dual Luciferase^®^ Reporter Assay System (Promega). Fusion proteins were assayed for interaction in all combinations.

### 2.6 Caspase 3/7 activity assays

Both wt and patient derived lymphoblasts were seeded at a concentration of 300,000 cells/ml of RPMI complete medium and treated with a concentration of 5 μg/ml of tunicamycin for 24 h. Samples were collected at indicated time points and mixed with equal parts Promega Caspase-Glo 3/7 reagent (Promega G8090) and incubated for 45 min. Luciferase activity was measured and compared to cell culture medium alone and untreated cells as the negative controls. To address the effect of inhibiting PACT-PKR interaction on cell viability, we cultured wt and patient lymphoblasts as described above in 50 µM of luteolin for 24 h followed by treatment with 5 μg/ml of tunicamycin in luteolin free media over the same 24 h.

### 2.7 RNA isolation and qRT-PCR

Total RNA was isolated from lymphoblasts using RNAzol RT (Sigma-Aldrich). After two washes with ice-cold PBS, 250 µL of RNAZol RT was added and total RNA was isolated as per the manufacturer’s instructions. For each sample we reverse transcribed with 800 ng of RNA using kit iScript™ Reverse Transcription Supermix for RT-qPCR (Bio-Rad, Hercules, CA, United States). The expression analysis of ATF4, CHOP and GAPDH was performed using the following primers.

ATF4 (Origene): Forward *5′-*TTC​TCC​AGC​GAC​AAG​GCT​AAG​G-*3*’

Reverse *5′-*CTC​CAA​CAT​CCA​ATC​TGT​CCC​G*-3’.*

CHOP (Origene): Forward *5′-*GGT​ATG​AGG​ACC​TGC​AAG​AGG​T*-3*’

Reverse *5′-*CTT​GTG​ACC​TCT​GCT​GGT​TCT​G*-3*’.

GAPDH (Origene): Forward *5′-*GTC​TCC​TCT​GAC​TTC​AAC​AGC​G-3’

Reverse 5′-ACC​ACC​CTG​TTG​CTG​TAG​CCA​A-3’

TaqMan Universal PCR Master Mix (Applied Biosystems), and cDNA derived from 40 ng total RNA was used. All reactions were run on a BioRad CFX96 Real-Time System C1000 thermal cycler machine using the conditions recommended for the primer sets (Origene). For each treated sample, relative quantification (RQ) (2^−ΔΔCT^) (Pfaffl, 2001), i.e., the normalized fold change relative to the mean of each of the controls, was calculated.

### 2.8 Data sharing

All data is contained within this manuscript. Data sharing is not relevant for this work.

## 3 Results

### 3.1 PKR is hyperactive in DYT-*PRKRA* cells sensitizing them to ER stress

**FIGURE 1:**
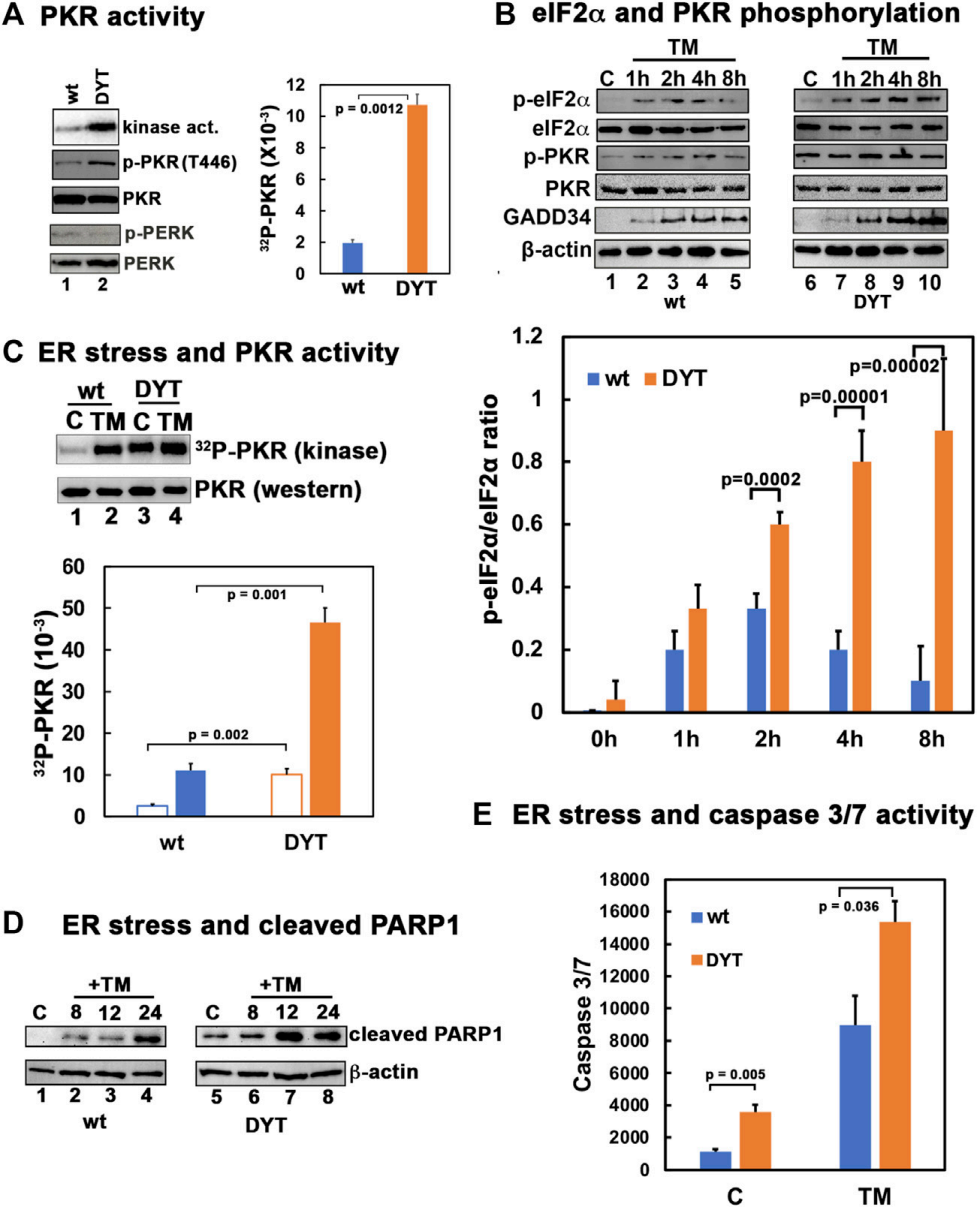
**(A)** PKR is hyperactive in DYT-*PRKRA* patient lymphoblasts. PKR activity assay and western blot analysis for p-PKR and total PKR. PKR kinase activity assay was performed using PKR immunoprecipitated from wt and DYT-*PRKRA* lymphoblast extracts using a monoclonal PKR antibody (R&D Systems) and protein A-sepharose beads. PKR activity was assessed without any externally added activator and the bands represent endogenous activity levels of PKR. The PKR band intensities were quantified using Imagequant TL (Cytiva), and the bar graph shows data from 3 independent experiments and the *p* values are as indicated. Blue bar: wt and orange bar: DYT-*PRKRA*. Whole cell extracts from normal (wt) and DYT-*PRKRA* patient derived lymphoblasts were analyzed. Blots were probed for p-PKR, total PKR, p-PERK, and total PERK. Best of three representative blots are shown. **(B)** Western blot analysis for p-PKR, p-eIF2α and GADD34. Normal (wt) and DYT-*PRKRA* patient derived lymphoblasts treated with 5 μg/ml of tunicamycin (TM) and cell extracts were prepared at various time points as indicated above the lanes after treatment and from untreated cells. Western blot analysis was performed with the indicated antibodies. The signal intensities of p-eIF2α and total eIF2α bands were quantified using Imagequant TL (Cytiva) and the ratio p-eIF2α/eIF2α was calculated for each time point using three separate experiments. The *p* values are as indicated. Blue bar: wt and orange bar: DYT-*PRKRA*. **(C)** PKR activity in wt and DYT-*PRKRA* cells after ER stress. Lymphoblast lines established from wt and DYT-*PRKRA* patient were treated with 5 μg/ml tunicamycin and cells extracts were prepared for PKR kinase activity assay and western blot analysis 2 h after the treatment. PKR kinase activity assay was performed using immunoprecipitated PKR as in part A. The bar graph shows data from 3 independent experiments and the *p* values are as indicated. Blue bar: wt and orange bar: DYT-*PRKRA*. Whole cell extracts from normal (wt) and DYT-*PRKRA* patient derived lymphoblasts were analyzed for total PKR. **(D)** Western blot analysis for cleaved PARP1. Whole cell extracts from normal (wt) and DYT-PRKRA patient derived lymphoblasts treated with 5 μg/ml of tunicamycin (TM) were analyzed at indicated time points using anti-cleaved PARP1 and anti-β-actin antibodies. **(E)** Caspase-Glo 3/7 activity. Lymphoblast lines established from wt and DYT-PRKRA patient were treated with 5 μg/ml tunicamycin and the caspase 3/7 activities were measured at 0 h and 24 h. Blue bars: wt cells, and orange bars: DYT-*PRKRA* cells. The data is an average of three independent experiments and the *p* values are as indicated.

Previously, our research established that DYT-*PRKRA* patient lymphoblasts are more susceptible to ER stress compared to the unaffected, wild type (wt) lymphoblasts (Vaughn et al., 2015; Burnett et al., 2020). To investigate if this susceptibility to apoptosis results from higher levels of PKR’s kinase activity, we performed a PKR activity assay to measure active kinase levels and a western blot analysis to compare levels of the phosphorylated form of PKR (p-PKR) in wt and patient cells in the absence of any stress. As seen in Figure 1A, the DYT-*PRKRA* patient cells show about 5-fold higher levels of PKR kinase activity (orange bar) compared to the wt cells (blue bar) in the absence of ER stress. The higher levels of active PKR were further supported by the western blot analysis with an antibody specific for p-PKR. These results demonstrate that DYT-*PRKRA* cells exhibit constitutive activation of PKR in the absence of ER stress. As PKR like endoplasmic reticulum resident kinase (PERK) is the other kinase that is activated in response to ER stress, we investigated if the total expression levels of PERK or phosphorylated active PERK were more in DYT-*PRKRA* cells. The levels of total PERK and phosphorylated form of PERK are similar in wt and DYT-*PRKRA* cells. As seen in Figure 1B, when subjected to ER stressor tunicamycin, the levels of eIF2α phosphorylation rise within 1 h in both wt and patient lymphoblasts. However, the patient lymphoblasts show significantly higher levels of eIF2α phosphorylation which also persists at 8 h after tunicamycin treatment whereas in wt cells there is a decrease in eIF2α phosphorylation at 8 h. PKR activation and levels of phosphorylated PKR also rise at 1 h after tunicamycin treatment and start to decline at 8 h after treatment in wt cells. In contrast, the levels of phosphorylated PKR are significantly high in the absence of treatment in the DYT-*PRKRA* patient cells with barely a detectable increase after tunicamycin treatment as analyzed by western blot analysis. The levels of GADD34, which is the regulatory subunit of protein phosphatase 1 (PP1) whose expression is induced in response to ER stress and acts to regulate the dephosphorylation of eIF2α and return cells to homeostasis were also compared in the wt and DYT-*PRKRA* cells. As seen, GADD34 is induced at higher levels in DYT-*PRKRA* cells as compared to wt cells. However, this increased expression of GADD34 is not sufficient to reduce the eIF2α phosphorylation that results from PKR activity remaining high at 8 h after ER stress in DYT-*PRKRA* cells. To confirm PKR activation in response to tunicamycin in DYT-*PRKRA* patient cells, we next performed PKR activity assays, which are more quantifiable and sensitive than the western blot analysis to detect a tunicamycin-induced increase in PKR activity above the high constitutive levels of activated PKR. As seen in Figure 1C, there is an increase in PKR activity following tunicamycin treatment in both wt and DYT-*PRKRA* patient lymphoblasts and the patient lymphoblasts have about 5-fold higher PKR activity as compared to wt lymphoblasts both with and without tunicamycin treatment. The elevated PKR kinase activity predisposes the DYT-*PRKRA* lymphoblasts to apoptosis as seen in Figures 1D, E. The levels of cleaved PARP1, which is a marker for apoptosis, are significantly higher in DYT-*PRKRA* patient lymphoblasts as compared to the wt lymphoblasts at 8–12 h after tunicamycin treatment. The levels of caspase 3/7 activity, another marker for apoptosis, are also significantly higher in DYT-*PRKRA* patient lymphoblasts in untreated as well as at 24 h after tunicamycin treatment (orange bars). These results thus indicate that the DYT-*PRKRA* lymphoblasts have elevated levels of active PKR at basal levels which increase further after ER stress.

### 3.2 Inhibition of PKR protects DYT-*PRKRA* cells against ER stress-induced apoptosis

**FIGURE 2:**
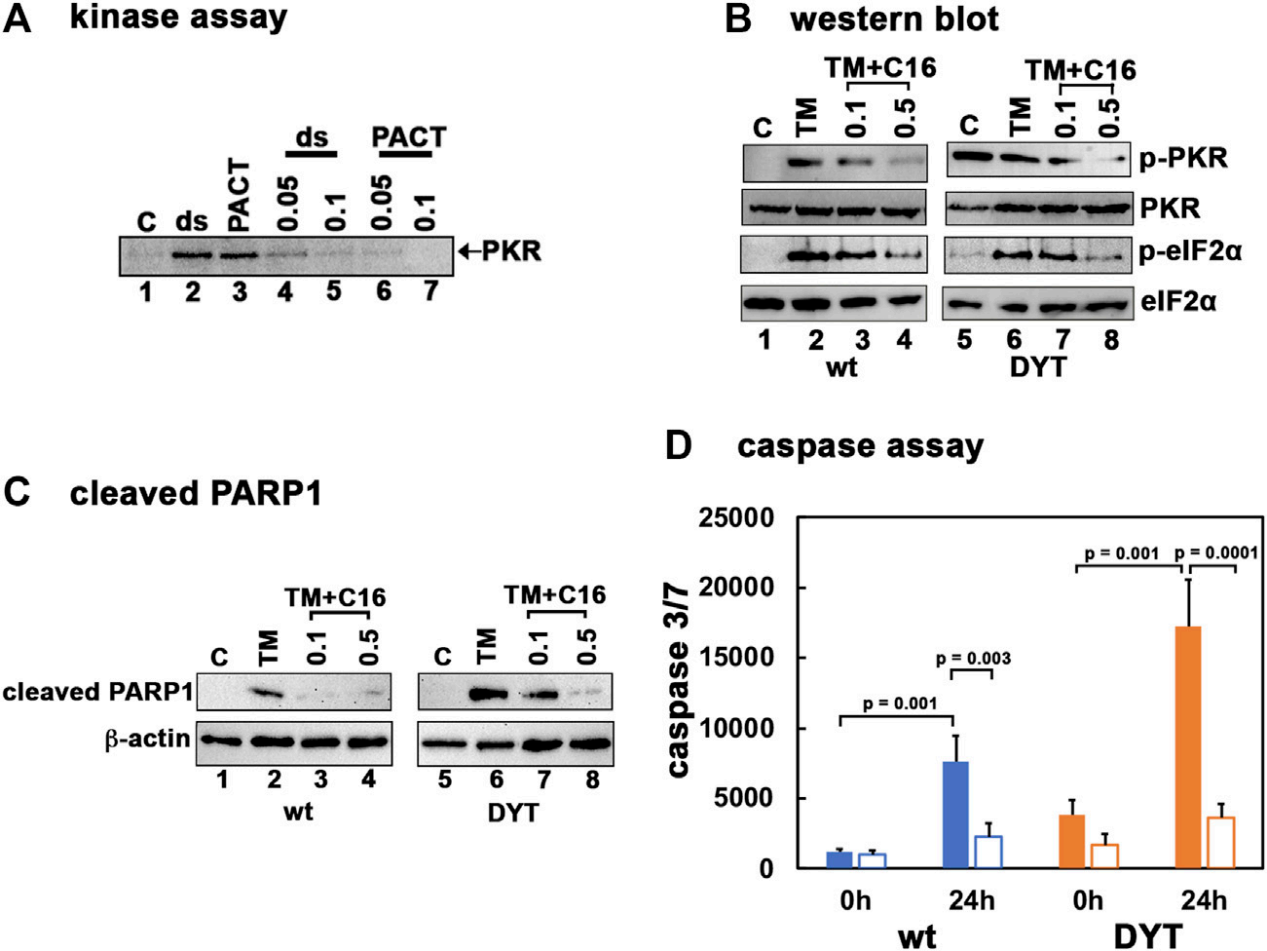
**(A)** PKR inhibition by C16. Kinase activity assay was performed using PKR immunoprecipitated from HeLa cell extracts using a monoclonal PKR antibody (R&D Systems) and protein A-sepharose beads. Either 1 μg/ml polyI:polyC (lanes 2, 4, 5) or 4 ng recombinant wt PACT (lanes 3, 6, 7) were used as PKR activators. C16 was added either at 0.05 μM (lanes 4 and 6) or 0.1 μM (lanes 5 and 7) as indicated on the top of the lanes. Lanes 1: no activator added. **(B)** Inhibition of PKR activation and eIF2α phosphorylation in lymphoblasts by C16. The normal (wt) and DYT-*PRKRA* patient derived lymphoblasts were treated with either 5 μg/ml of tunicamycin (TM), TM + 0.1 μM C16 or TM + 0.5 μM C16 for 2 h. For the C16 treated samples, the cells were pretreated with C16 for 24 h before tunicamycin treatment. Whole cell extracts were prepared at 2 h after the tunicamycin treatment and were analyzed by western blot analysis. Blots were probed for p-PKR, total PKR, p-eIFα, and total eIF2α. Best of four representative blots are shown. **(C)** Western blot analysis for cleaved PARP1. The normal (wt) and DYT-PRKRA patient derived lymphoblasts were treated with either 5 μg/ml of tunicamycin (TM), TM + 0.1 μM C16 or TM + 0.5 μM C16 for 24 h. Whole cell extracts prepared at 24 h after treatments were analyzed using anti-cleaved PARP1 and anti-β-actin antibodies. **(D)** Caspase-Glo 3/7 activity. Lymphoblast lines established from wt and DYT-*PRKRA* patient were treated either with 5 μg/ml tunicamycin or with tunicamycin and 0.5 μM C16 for 24 h. The caspase 3/7 activities were measured at 0 h and 24 h. Blue bars: wt cells, and orange bars: DYT-PRKRA cells, filled bars: tunicamycin treated and unfilled bars: tunicamycin and C16 treated. The data is an average of three independent experiments and the *p* values are as indicated.

To test if inhibition of PKR activity can protect the DYT-*PRKRA* cells from ER stress-induced apoptosis, we used an established PKR inhibitor C16 (Jammi et al., 2003; Ingrand et al., 2007; Tronel et al., 2014; Xiao et al., 2016; Li et al., 2017; Farabaugh et al., 2020; Li et al., 2020; Watanabe et al., 2020). Our previous results established that the PACT mutations in DYT-*PRKRA* patients cause enhanced association of PACT with PKR in the absence of stress and result in elevated PKR activation (Burnett et al., 2020). The enhanced PKR activation observed in DYT-*PRKRA* lymphoblasts (Figure 1) thus results from PACT-mediated PKR activation, making it important to determine that C16 inhibits PKR when activated by PACT. Previously, C16 was reported to inhibit PKR when activated by PACT (Farabaugh et al., 2017; Farabaugh et al., 2020) and thus we first confirmed this in DYT-*PRKRA* cells. As seen in Figure 2A, in the absence of an activator, PKR activity is barely detectable (lane 1) and dsRNA (lane 2), as well as PACT (lane 3), both activate PKR robustly. When added in the presence of dsRNA or PACT, C16 inhibits PKR significantly at both concentrations tested (lanes 4–7). Next, we tested the actions of C16 on PKR activity in wt and DYT-*PRKRA* patient lymphoblasts. As seen in Figure 2B, tunicamycin treatment activated PKR strongly in wt cells (lane 2) and this activation is inhibited significantly at 0.1 μM and almost completely at 0.5 μM of C16 (lanes 3 and 4). Similarly, in the DYT-*PRKRA* patient lymphoblasts, PKR activity is partially inhibited at 0.1 μM and almost completely at 0.5 μM of C16 (lanes 7 and 8). The effect of C16 on eIF2α phosphorylation seems less pronounced compared to its effect on PKR, possibly because C16 does not inhibit PERK. The eIF2α phosphorylation is significantly reduced in both wt and DYT-*PRKRA* cells by 0.5 μM of C16 (lanes 4 and 8). To investigate the effect of C16 on apoptosis induced by tunicamycin, we used both the cleaved PARP1 and caspase assays. As seen in Figure 2C, in wt lymphoblasts, C16 inhibited PARP1 cleavage significantly at both 0.1 and 0.5 μM concentrations (lanes 3 and 4). In DYT-*PRKRA* patient lymphoblasts, C16 inhibited PARP1 cleavage partially at 0.1 μM (lane 7) and almost completely at 0.5 μM (lane 8). To further confirm that C16 can inhibit apoptosis, we used a caspase 3/7 assay. As seen in Figure 2D, DYT-*PRKRA* patient lymphoblasts (orange bars) show a higher level of caspase activity without any ER stress and this basal caspase activity is inhibited by C16. At 24 h after tunicamycin treatment, the caspase activity increases about 6-fold in wt (blue bars) and about 4.5-fold in DYT-*PRKRA* cells. C16 inhibits this increase significantly in both wt and DYT-*PRKRA* cells with about 70% decrease in wt (blue bars) and about 80% decrease in DYT-*PRKRA* cells (orange bars). These results establish that inhibition of PKR protects both wt and DYT-*PRKRA* cells after ER stress.

### 3.3 Luteolin disrupts the stronger PACT-PKR interaction in DYT-*PRKRA* cells

**FIGURE 3:**
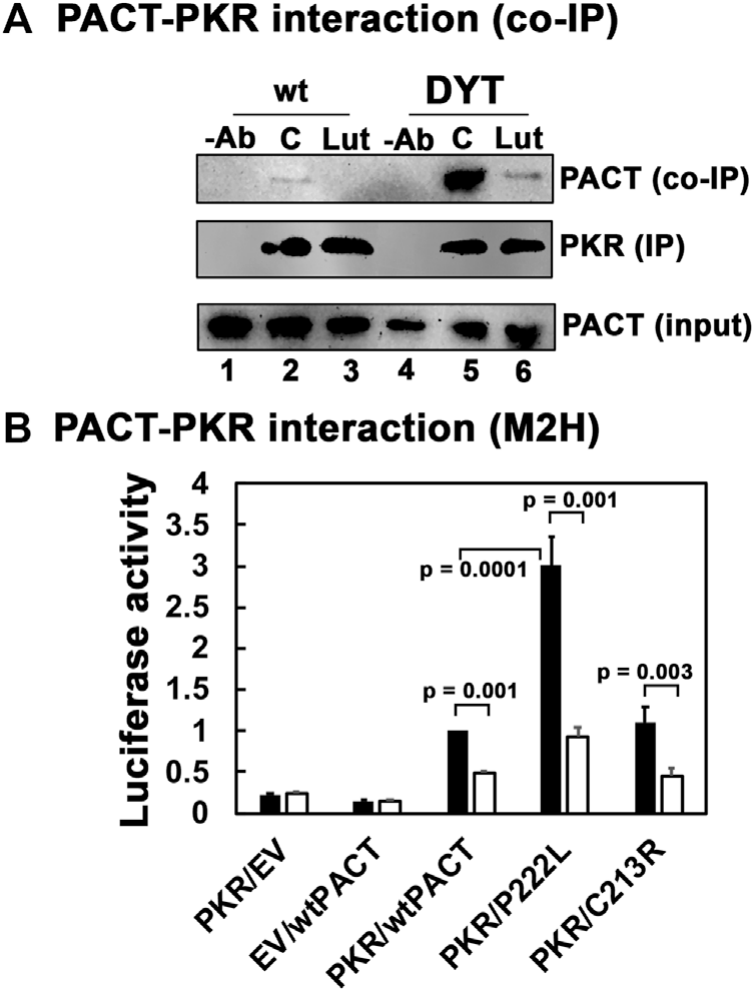
**(A)** Luteolin disrupts the interaction between PKR and PACT. **(A)** Co-IP of endogenous PKR and PACT proteins. Lymphoblasts from unaffected family member (wt) or DYT-PRKRA patient (patient) were treated with 50 μM luteolin. The cell extracts were prepared 24 h after the treatment, and endogenous PKR protein was immunoprecipitated using anti-PKR mAb and protein A-sepharose, which immunoprecipitates total PKR. The immunoprecipitates were analyzed by western blot analysis with anti-PACT monoclonal antibody (Co-IP panel). The blot was stripped and re-probed with anti-PKR mAb to ascertain an equal amount of PKR was immunoprecipitated in each lane (IP panel). Input blot: Western blot analysis of total proteins in the extract with anti-PACT mAb showing equal amount of PACT in all samples. **(B)** Mammalian two-hybrid analysis. HeLa cells were transfected with 250 ng of each of the two test plasmids encoding proteins to be tested for interaction, 50 ng of the reporter plasmid pG5Luc, and 1 ng of plasmid pRL-Null to normalize transfection efficiency. 2 h after transfection, one set of samples were left untreated, and one set was treated with 50 μg/ml luteolin. Cells were harvested 24 h after luteolin treatment, and cell extracts were assayed for luciferase activity. The plasmid combinations are as indicated, PKR was expressed as a GAL4 DNA-binding domain fusion protein (bait) and all PACT proteins were expressed as VP16-activation domain fusion proteins (preys). The experiment was repeated twice with each sample in triplicate, and the averages with standard error bars are presented. The *p* values are as indicated. RLU, relative luciferase units.

Previously, we have established that luteolin, a plant flavonoid, disrupts the interaction between PACT and PKR (Dabo et al., 2017; Burnett et al., 2020). In human THP-1 macrophages, luteolin inhibits PKR phosphorylation and the induction of pro-inflammatory cytokines in response to oxidative stress and toll-like receptor (TLR) agonist lipopolysaccharide (Dabo et al., 2017). The ISR induced by oxidative stress or ER stressor thapsigargin was only partially blocked by luteolin treatment in this study, which was attributed to the activity of PERK remaining unaffected by luteolin. In our DYT-PRKRA cells, we wanted to characterize if luteolin can effectively disrupt the enhanced interaction between PACT mutant P222L and PKR. To determine this, we used coimmunoprecipitation analysis with wt and DYT-*PRKRA* patient lymphoblasts that are homozygous for P222L mutation (Vaughn et al., 2015). We have established previously that luteolin disrupts the PACT-PKR interaction in compound heterozygous DYT-*PRKRA* lymphoblasts carrying P222L and C213R mutations (Burnett et al., 2020). We used the P222L homozygous lymphoblasts in the coimmunoprecipitation analysis because we have previously established that in compound heterozygous patient cells, only the P222L-PKR interaction is enhanced but the C213R-PKR interaction has similar affinity as the wt PACT-PKR interaction (Burnett et al., 2020). Both P222L homozygous and compound heterozygous DYT-*PRKRA* cells undergo enhanced apoptosis in response to ER stress (Vaughn et al., 2015; Burnett et al., 2020) and thus the P222L homozygous cells are better suited for coimmunoprecipitation analysis without any interference from the C213R mutant that would occur in the compound heterozygous patient cells. As seen in Figure 3A, in the absence of any ER stress, the wt lymphoblasts show very slight interaction between PACT and PKR (lane 2, co-IP panel), which is characteristic in the absence of a stress signal and in accordance with previous research (Vaughn et al., 2015). However, the DYT-*PRKRA* cells homozygous for P222L mutation show markedly enhanced interaction between PKR and PACT (lane 5, co-IP panel) even in the absence of ER stress. When treated with luteolin for 24 h, the interaction between PACT and PKR in wt lymphoblasts is undetectable (lane 3, co-IP panel), and the interaction between P222L mutant and PKR is markedly reduced (lane 6, co-IP panel) indicating that luteolin disrupts the enhanced interaction between P222L mutant and PKR. The IP panel shows that an equal amount of PKR was immunoprecipitated in all samples except for antibody-negative controls (lanes 1 and 4). The input panel shows that equal amounts of PACT were present in all samples. These results establish that a 24 h treatment with luteolin disrupts the PACT-PKR interaction in DYT-*PRKRA* patient cells. To confirm these results further, we tested the interaction between PACT and PKR using mammalian two-hybrid analysis. We have previously used such analyses to establish that the DYT-*PRKRA* mutations result in enhanced interactions between PACT and PKR in intact mammalian cells in the absence of a stress signal (Vaughn et al., 2015; Burnett et al., 2020). As seen in Figure 3B, the PKR interaction with wt PACT is detectable at basal levels in the absence of ER stress in this system and luteolin treatment disrupts this interaction significantly (white bars). As compared to this, the interaction between P222L mutant and PKR is about 3-fold stronger at basal levels in the absence of stress and luteolin can disrupt the interaction markedly. The C213R-PKR interaction is comparable to the wt PACT-PKR interaction as expected based on our previous research (Burnett et al., 2020) and is also disrupted efficiently by luteolin. These results establish that luteolin disrupts the stronger interaction between DYT-*PRKRA* mutant P222L and PKR and indicated that luteolin may potentially be a good candidate to test for protecting the DYT-*PRKRA* cells from ER stress-induced apoptosis.

### 3.4 Luteolin protects DYT-PRKRA cells from ER stress-induced apoptosis

**FIGURE 4:**
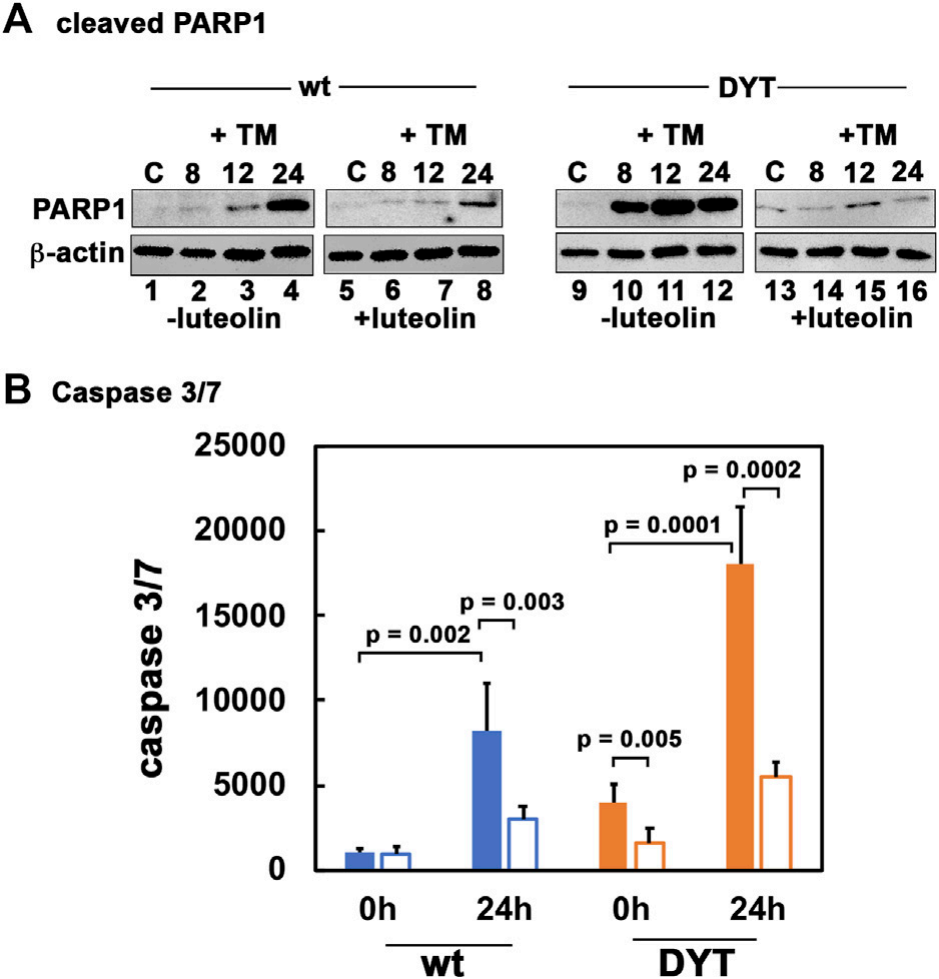
Luteolin protects DYT-*PRKRA* cells from apoptosis in response to ER stress. **(A)** Western blot analysis for cleaved PARP1. The normal (wt) and DYT-*PRKRA* patient derived lymphoblasts were treated with 5 μg/ml of tunicamycin (TM) either without pretreatment with luteolin or with 50 µM luteolin pretreatment for 24 h. Whole cell extracts prepared at the indicated time points after TM treatment were analyzed using anti-cleaved PARP1 and anti-β-actin antibodies. **(B)** Caspase-Glo 3/7 activity. The normal (wt) and DYT-*PRKRA* patient derived lymphoblasts were treated with 5 μg/ml of tunicamycin (TM) either without pretreatment with luteolin or with 50 µM luteolin pretreatment for 24 h. The caspase 3/7 activities were measured at 0 h and 24 h. Blue bars: wt cells, and orange bars: DYT-*PRKRA* cells, filled bars: tunicamycin treated and unfilled bars: luteolin and tunicamycin treated. The data is an average of three independent experiments and the *p* values are as indicated.

We next tested the ability of luteolin to protect DYT-*PRKRA* cells from ER stress-induced apoptosis using PARP1 cleavage and caspase 3/7 activity as apoptosis markers. As seen in Figure 4A, in the absence of luteolin pre-treatment, there are significant amounts of cleaved PARP1 at 12 and 24 h after tunicamycin treatment in wt cells (lanes 3 and 4), which is markedly reduced by luteolin pre-treatment (lanes 7 and 8). In contrast to wt cells, the DYT-*PRKRA* cells show markedly increased cleaved PARP1 at 8, 12, and 24 h after tunicamycin treatment (lanes 10–12) and luteolin pre-treatment significantly reduces the amount of cleaved PARP1 at all these time points after tunicamycin treatment (lanes 14–16). In agreement with this, as seen in Figure 4B, there is a significant reduction of caspase 3/7 activity after ER stress in luteolin pre-treated cells. The wt cells show about 7.5-fold induction of caspase 3/7 activity at 24 h after tunicamycin treatment, and luteolin pre-treatment shows about 64% repression (blue bars). Compared to wt cells, the DYT-*PRKRA* patient cells, there is about 4-fold higher level of caspase 3/7 activity in the absence of any stressor, and luteolin can repress about 60% of this basal activity (orange bars). The DYT-*PRKRA* patient cells show about 4.5-fold induction of caspase3/7 activity 24 after tunicamycin treatment and luteolin pre-treatment shows about 70% reduction, thus supporting the PARP1 cleavage results in Figure 3A. Luteolin is thus effective in protecting both the higher basal level of apoptosis in DYT-*PRKRA* cells as well as tunicamycin-induced apoptosis in both wt and DYT-*PRKRA* cells.

### 3.5 Luteolin suppresses higher PKR and eIF2α hyperphosphorylation in DYT-*PRKRA* cells

**FIGURE 5:**
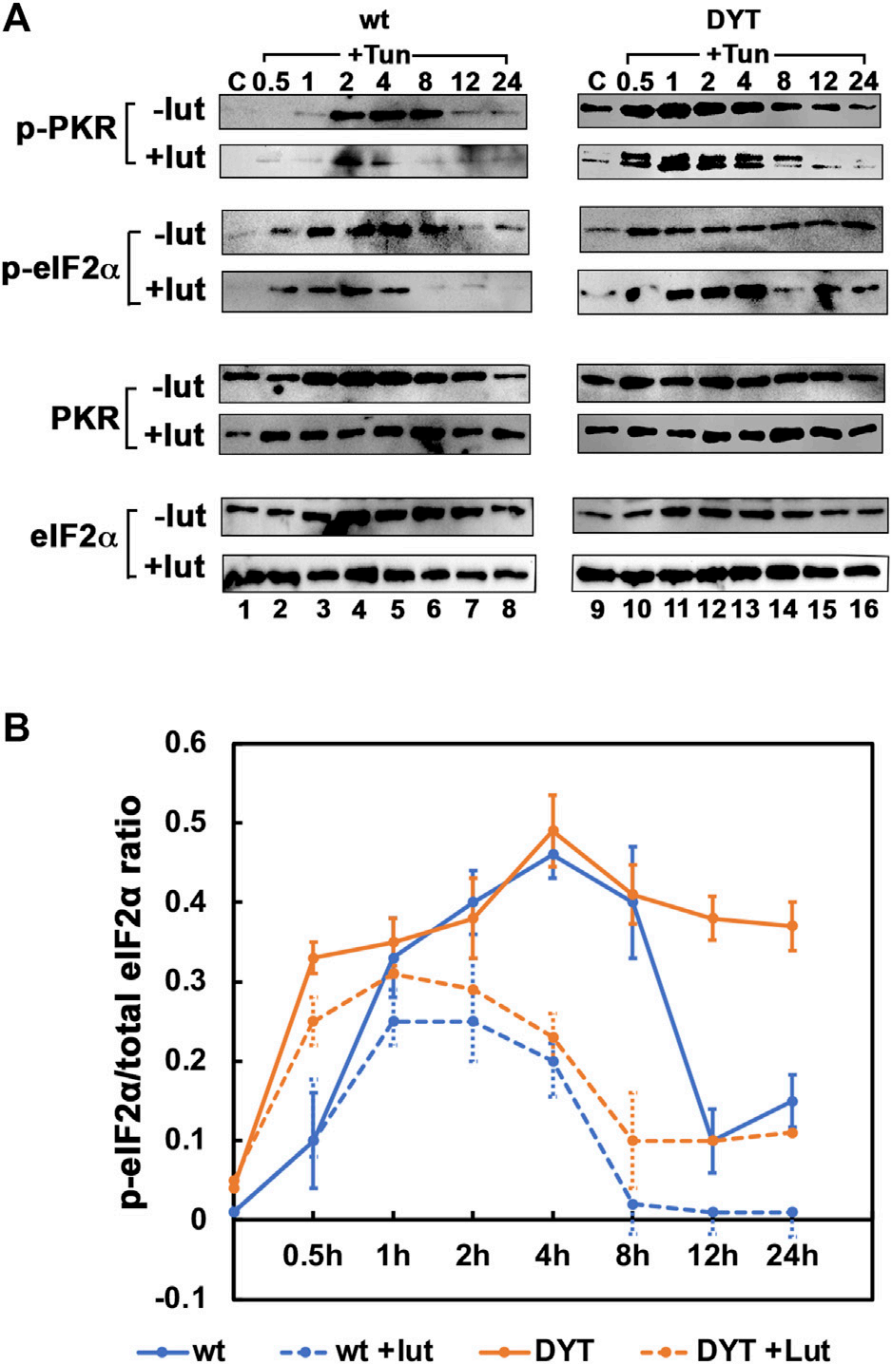
**(A)** Effect of luteolin on PKR activation and ISR in response to tunicamycin in normal and DYT-*PRKRA* patient lymphoblasts: western blot analysis for p-PKR and p-eIF2α. Whole cell extracts from normal (wt) and DYT-PRKRA patient derived lymphoblasts treated with 5 μg/ml of tunicamycin (TM) without any luteolin pretreatment or after 24 h pretreatment with 50 µM luteolin were analyzed at indicated time points. Blots were probed for p-eIF2α, total eIF2α, p-PKR, and total PKR. Best of four representative blots are shown. **(B)** The signal intensities of p-eIF2α and total eIF2α bands were quantified using Imagequant TL (Cytiva) and the ratio p-eIF2α/eIF2α was calculated for each time point using four separate experiments. The *p* values for differences between − lut and + lut for both wt and DYT-*PRKRA* cells were all below 0.001. Blue lines: wt and orange lines: DYT-*PRKRA*. Solid lines: without luteolin and dotted lines: with luteolin.

To further assess the effect of luteolin on the PKR activation and eIF2α phosphorylation and understand the mechanism for the protection from apoptosis offered by luteolin, we pre-treated the wt and DYT-*PRKRA* patient cells with luteolin for 24 h and then treated with tunicamycin for various time intervals to compare their response. As seen in Figure 5, tunicamycin treatment induced significant PKR phosphorylation at 2, 4, and 8 h after the treatment in wt cells (-lut panel, lanes 4–6) and the luteolin pre-treatment reduced both the level of PKR phosphorylation and the duration (+lut panel, lanes 4–6). In DYT-*PRKRA* cells, tunicamycin treatment induced a detectable PKR phosphorylation above the high basal level (-lut panel, lanes 10–14), and the luteolin pre-treatment reduced both the level of PKR phosphorylation and the duration (+lut panel, lanes 10–14). In agreement with this, the eIF2α phosphorylation levels and duration are also significantly reduced after luteolin treatment in both wt and DYT-*PRKRA* cells (compare p-eIF2α: − lut and +lut panels, lanes 2–6 and lanes 10–14). We quantified the band intensities of p-eIF2α and total eIF2α from four independent experiments and calculated the ratio of p-eIF2α to total eIF2α, which is represented in a graphical format in Figure 5B. The results indicate that disrupting the interaction between PACT and PKR blunts the level and duration of both PKR and eIF2α phosphorylation in wt and DYT-*PRKRA* patient cells. This indicates that luteolin-mediated protection of the DYT-*PRKRA* cells after ER stress could result from inhibition of PKR activity. Thus, the excessive or prolonged phosphorylation of PKR and eIF2α is prevented by luteolin and this may be one of the reasons for restoration of homeostasis after ER stress.

### 3.6 Luteolin inhibits ER stress-induced expression of ATF4 and CHOP

**FIGURE 6:**
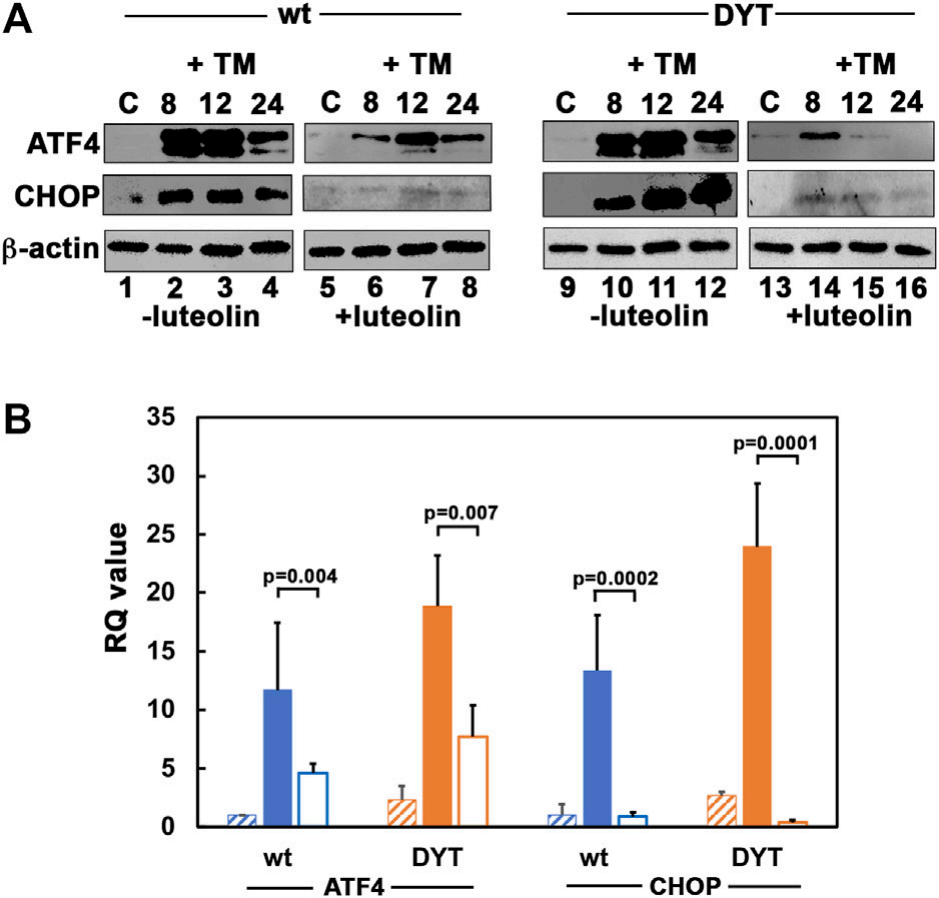
**(A)** Effect of luteolin on PKR activation and ISR in response to tunicamycin in normal and DYT-*PRKRA* patient lymphoblasts: western blot analysis for ATF4 and CHOP. Whole cell extracts from normal (wt) and DYT-*PRKRA* patient derived lymphoblasts treated with 5 μg/ml of tunicamycin (TM) without any luteolin pretreatment or after 24 h pretreatment with 50 µM luteolin were analyzed at indicated time points. Blots were probed for ATF4, and CHOP. Best of four representative blots are shown. β-actin was used as a loading control to ensure equal amounts of protein was loaded in each lane. **(B)** DYT-*PRKRA* patient lymphoblasts express higher levels of ATF4 and CHOP mRNAs in response to tunicamycin and luteolin treatment downregulates ATF4 and CHOP induction. Quantitative RT-PCR of ATF4 and CHOP in wt (blue bars) and DYT-*PRKRA* (orange bars) lymphoblasts. The hatched bars indicate untreated control values, solid filled bars indicate tunicamycin treated values, and unfilled bars indicate luteolin and tunicamycin treated values. The RQ values indicate that ATF4 and CHOP expression was upregulated in response to tunicamycin and this upregulation was suppressed by luteolin pre-treatment in both wt and DYT-*PRKRA* cells. Data from 3 separate experiments was analyzed and the *p* values are as indicated.

We next examined if the ER stress-dependent induction of transcription factors ATF4 and CHOP also reflect a similar reduction after luteolin treatment. As seen in Figure 6A, the DYT-*PRKRA* lymphoblasts induced ATF4 and CHOP at higher levels (lanes 9–12) as compared to wt lymphoblasts (lanes 1–4). Luteolin treatment attenuated both ATF4 and CHOP induction significantly in wt lymphoblasts (lanes 5–8) as well as in DYT-*PRKRA* lymphoblasts (lanes 13–16). While there was almost a complete block of CHOP induction in luteolin treated cells, ATF4 induction was significantly reduced by luteolin treatment. There was also a corresponding reduction in the mRNA levels of ATF4 and CHOP as seen in Figure 6B. This can partly explain the protection from apoptosis seen in Figure 4 as CHOP is known to contribute to apoptosis after ER stress (Silva et al., 2005; Sano and Reed, 2013). CHOP has been shown to induce genes involved in protein synthesis (Han et al., 2013) and high rates of protein synthesis leads to ATP depletion, oxidative stress, and cell death, thus high levels of expression of CHOP are known to be harmful for cellular recovery and homeostasis after ER stress.

### 3.7 Luteolin inhibits the persistent PACT-PKR interaction at later time points in DYT-*PRKRA* cells while allowing transient PACT-PKR interaction at earlier time points after ER stress

**FIGURE 7:**
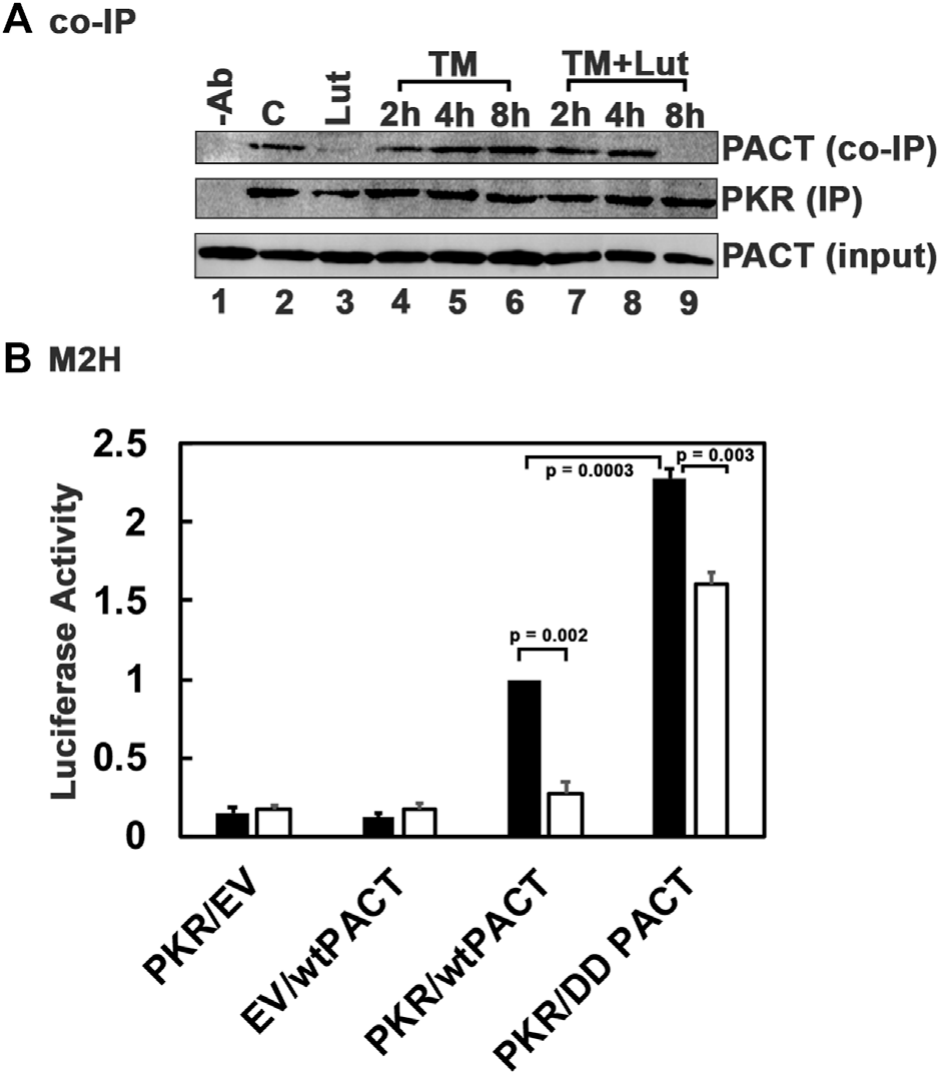
Luteolin allows for a transient PACT-PKR interaction after ER stress. **(A)** Co-IP of endogenous PKR and PACT proteins. The DYT-PRKRA lymphoblasts were treated with 5 μg/ml tunicamycin (TM) either with or without luteolin pre-treatment for 24 h. The cell extracts were prepared at indicated time points after the tunicamycin treatment, and endogenous PKR protein was immunoprecipitated using anti-PKR mAb and protein A-sepharose, which immunoprecipitates total PKR. The immunoprecipitates were analyzed by western blot analysis with anti-PACT monoclonal antibody (Co-IP panel). The blot was stripped and re-probed with anti-PKR mAb to ascertain an equal amount of PKR was immunoprecipitated in each lane (IP panel). Input blot: Western blot analysis of total proteins in the extract with anti-PACT mAb showing equal amount of PACT in all samples. **(B)** Mammalian two-hybrid analysis. HeLa cells were transfected with 250 ng of each of the two test plasmids encoding proteins to be tested for interaction, 50 ng of the reporter plasmid pG5Luc, and 1 ng of plasmid pRL-Null to normalize transfection efficiency. 2 h after transfection, one set of samples were left untreated and one set was treated with 50 µM luteolin. Cells were harvested 24 h after luteolin treatment, and cell extracts were assayed for luciferase activity. The plasmid combinations are as indicated, PKR was expressed as a GAL4 DNA-binding domain fusion protein (bait) and all PACT proteins were expressed as VP16-activation domain fusion proteins (preys). The experiment was repeated twice with each sample in triplicate, and the averages with standard error bars are presented. The *p* values are as indicated. RLU, relative luciferase units.

Our previous work established that PACT is phosphorylated in response to stress signals and the phosphorylated PACT associates with PKR at a higher affinity thereby inducing PKR activation (Patel et al., 2000; Singh et al., 2009; Singh et al., 2011). As luteolin disrupts the interaction between PACT and PKR, we reasoned that luteolin may be able to disrupt the enhanced interaction between mutant PACT and PKR present in DYT-*PRKRA* cells in the absence of ER stress while permitting a transient interaction of phosphorylated mutant PACT with PKR at early time points after ER stress. This would explain why PKR can still show activation after ER stress in the presence of luteolin (Figure 5). We tested this using coimmunoprecipitation and a mammalian two-hybrid analysis. As seen in Figure 7A, in DYT-*PRKRA* cells, mutant PACT coimmunoprecipitates with PKR efficiently in the absence of ER stress (lane 2). A luteolin treatment for 24 h results in a complete disruption of PACT-PKR interaction and no co-immunoprecipitation of mutant PACT can be detected (lane 3). The PACT-PKR interaction is maintained after tunicamycin treatment in the absence of luteolin (lanes 4–6). Interestingly, when treated with tunicamycin to induce ER stress after a 24 h pretreatment with luteolin, mutant PACT co-immunoprecipitates with PKR is at 2 h and 4 h after tunicamycin treatment (lanes 7 and 8) but not at 8 h after tunicamycin treatment (lane 9). As 24 h after luteolin treatment PACT-PKR interaction is significantly disrupted (lane 3), these results indicate that early phosphorylation of PACT after tunicamycin treatment (Singh et al., 2009) allows for PACT-PKR interaction in the presence of luteolin but at later time points when PACT-PKR interaction is disrupted. These results demonstrate that luteolin disrupts the high PACT-PKR interaction very efficiently in the absence of ER stress when mutant PACT is not phosphorylated. However, once phosphorylated after ER stress (Singh et al., 2009), the stronger interaction between phosphorylated mutant PACT and PKR can occur in the presence of luteolin only while PACT stays phosphorylated (lane 9). This was further tested using a mammalian two-hybrid interaction assay and a phosphomimic mutant of PACT where we replaced the two serines at 246 and 287 that are phosphorylated in after stress signals with aspartic acids (S246D, S287D or DD mutant). This mutant has been used previously in several studies by us and other labs as it is established that the phosphorylation of serines 246 and 287 results in enhanced interaction between PACT and PKR after cellular stress (Peters et al., 2006; Singh et al., 2011; Singh and Patel, 2012). As seen in Figure 7B, PKR and wt PACT interact at a detectable level in this assay and the interaction is enhanced more than 2-fold between the phosphomimic mutant PACT (DD PACT) as indicated by the black bars. When treated with luteolin, the interaction between PKR and wt PACT is barely detectable above the negative controls but the interaction between DD PACT and PKR is still detectable (white bars), although reduced compared to the interaction in the absence of luteolin. These results indicate that luteolin prevents the enhanced PACT-PKR interactions in DYT-PRKRA cells at the late adaptive phase of ISR and allows restoration of cellular homeostasis while maintaining the PKR and eIF2α phosphorylation at the earlier time points after ER stress.

## 4 Discussion

To develop effective therapeutic strategies for dystonia, it is essential to understand the underlying molecular mechanisms that lead to this movement disorder. Aiming to elucidate the possible pathological mechanisms, our previous work on DYT-*PRKRA* focused on studying how the mutations reported in DYT-*PRKRA* patients affect the biological PKR activation function of PACT (Vaughn et al., 2015; Burnett et al., 2019; Burnett et al., 2020). As enhanced PKR activation due to stronger PACT-PKR interactions emerged as a common theme for DYT-*PRKRA*, in this study we examined the effect of disrupting PACT-PKR interactions using luteolin. Luteolin is a natural flavonoid that exhibits beneficial effects on human health, which have been described in several traditional medicines that make therapeutic use of natural plants, fruits, and herbs (Muruganathan et al., 2022). With the availability of modern analytical biochemical and molecular techniques, luteolin’s effects on a variety of cellular responses have been studied and documented and currently luteolin is being explored for its beneficial activity in treating various human ailments (Caporali et al., 2022). Among the diverse health benefits of luteolin, its anti-cancer, anti-microbial, anti-inflammatory, antioxidant, and anti-diabetic effects have been studied in detail in various cell types and mouse models (Muruganathan et al., 2022). Additionally, luteolin is blood-brain barrier permeable and is reported to have a neuroprotective effect in cell culture and animal models of Alzheimer’s (Wang et al., 2016), Parkinson’s (Siddique, 2021), and Huntington’s (Oliveira et al., 2015) disease. A combination of luteolin and quercetin also proved effective in reducing symptoms of autism spectrum disorders (ASD) (Taliou et al., 2013). The exact mechanisms by which luteolin exerts these effects remains poorly characterized and the effects are often thought to be pleiotropic. We previously identified luteolin as an inhibitor of the PKR-PACT interaction using high-throughput screening of chemical libraries (Dabo et al., 2017). Thus, our current study to test therapeutic potential of luteolin for DYT-*PRKRA* stemmed from the prior extensive biochemical and molecular knowledge about the disease mechanisms operative in DYT-*PRKRA* and the demonstrated ability of luteolin to disrupt PACT-PKR interactions.

**FIGURE 8:**
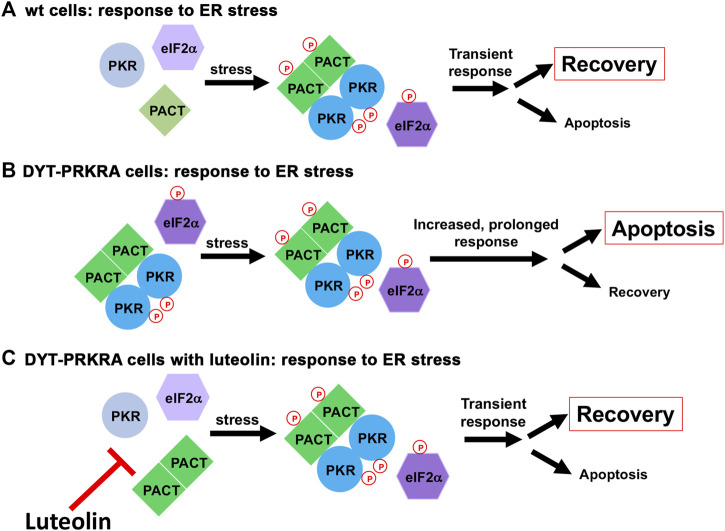
A schematic model for ER stress response in wt and DYT-*PRKRA* cells. **(A)** ER stress response in wt cells. In the absence of stress, PACT is not phosphorylated and PKR is not activated. After ER stress, PACT is phosphorylated and PACT-PACT and PACT-PKR interactions are enhanced thereby causing a transient PKR activation and eIF2α phosphorylation. This response leads to restoration of homeostasis promoting survival. **(B)** ER stress response in DYT-*PRKRA* cells. In the absence of stress, mutant PACT is not phosphorylated but forms strong PACT-PACT as well as PACT-PKR interactions and PKR is activated. After ER stress, PACT is phosphorylated and PACT-PACT and PACT-PKR interactions are further enhanced thereby causing a persistent PKR activation and eIF2α phosphorylation promoting apoptosis. **(C)** ER stress response in DYT-*PRKRA* cells in the presence of luteolin. In the absence of stress, mutant PACT is not phosphorylated and the PACT-PKR interactions are disrupted by luteolin and PKR is not activated. After ER stress, PACT is phosphorylated and PACT-PACT and PACT-PKR interactions are enhanced thereby causing a transient PKR activation and eIF2α phosphorylation. This transient response leads to restoration of homeostasis promoting survival.

Our results presented in this study indicate that disrupting PACT-PKR interactions in DYT-*PRKRA* patient cells represses PKR activation, eIF2α phosphorylation, ATF4 induction as well as CHOP induction in response to ER stress. Luteolin also prevented the higher levels of apoptosis seen in DYT-PRKRA cells in response to ER stress. Our results indicated that although luteolin disrupts the strong PACT-PKR interactions observed in patient cells in the absence of stress, it allows for the stress-induced and transient PACT-PKR interaction. PACT is phosphorylated constitutively at serine 246 in the absence of stress and is rapidly phosphorylated at serine 287 in response to cellular stress (Patel et al., 2000; Peters et al., 2006; Singh et al., 2011; Singh and Patel, 2012). Our results in Figure 7 demonstrated that luteolin does not disrupt the transient stress-dependent interaction between PACT and PKR. Thus, luteolin prevents enhanced PACT-PKR interactions in DYT-*PRKRA* patient cells in the absence of stress while preserving the normal stress-induced transient PACT-PKR interactions to allow for a transient PKR activation during ISR (Figure 8). This potentially indicates that the interaction between phosphorylated PACT and PKR has higher affinity than the affinity between DYT-*PRKRA* PACT mutants and PKR.

It is interesting that disrupting PACT-PKR interaction with luteolin almost completely prevents induction of CHOP, a transcription factor that contributes at least in part to apoptosis after ER stress (Zinszner et al., 1998; Oyadomari et al., 2002; Silva et al., 2005; Sano and Reed, 2013). Although the repression of CHOP induction after luteolin treatment supports previous research that reported an essential role of PACT-mediated PKR activation in ER stress-induced apoptosis (Lee et al., 2007; Singh et al., 2009), it is possible that the antioxidant actions of luteolin contribute to its protective effects after ER stress. Previously it has been observed that an antioxidant treatment and CHOP deletion act through a common mechanism to suppress apoptosis after ER stress (Malhotra et al., 2008). In future, the contribution of antioxidant actions of luteolin towards protection from apoptosis after ER stress needs to be examined by comparing the actions of luteolin with other antioxidants that have no effect on PACT-PKR interaction. Additionally, transcriptional induction by ATF4 and CHOP has also been shown to increase protein synthesis leading to oxidative stress and cell death (Han et al., 2013), thus indicating that CHOP may contribute to apoptosis *via* induction of oxidative stress. However, in our study, we observe the protective actions of luteolin in the absence of CHOP, as the CHOP induction is almost completely blocked after luteolin treatment. Any contribution of luteolin’s antioxidant actions towards CHOP induction after ER stress can be investigated in the future studies to understand the contribution of oxidative stress for CHOP induction. Furthermore, in our current study we did not investigate the effects of luteolin under conditions of chronic stress, which is likely to be present in DYT-PRKRA cells at basal low levels. As ATF4 and CHOP has been shown to contribute to a coordinated stress-induced transcriptional reprograming that prevents cell death under conditions of chronic ER stress (Guan et al., 2017), in future studies, luteolin’s effects on possible reprograming in DYT-*PRKRA* cells can offer mechanistic insights.

We have previously shown that PACT-induced PKR activation is essential for tunicamycin-induced apoptosis and PACT as well as PKR null cells are markedly resistant to apoptosis, show defective eIF2α phosphorylation and compromised CHOP induction (Singh et al., 2009). A reconstitution of PKR and PACT expression in the respective null cells rendered them sensitive to tunicamycin, thus establishing that PACT-induced PKR activation plays an essential function in induction of apoptosis. Additionally, when overexpression of the trans-dominant negative, catalytically inactive mutant K296R was used to inhibit PKR in neuroblastoma cells, it protected the cells from undergoing apoptosis (Vaughn et al., 2014). K296R overexpressing cells showed defective PKR activation, delayed eIF2α phosphorylation, compromised CHOP expression, and reduced caspase-3 activation.

Our approach of inhibiting the heightened PKR activation observed in DYT-*PRKRA* with luteolin while preserving a transient PKR activation under conditions of stress could be helpful for treatment of diseases that involve overactive PKR (Blalock et al., 2010). Higher levels of activated PKR are noted in post-mortem patient studies as well as in mouse models of neurodegenerative conditions (Marchal et al., 2014; Hugon et al., 2017; Gal-Ben-Ari et al., 2018). Increased levels of phosphorylated PKR have been reported in the brains of Alzheimer’s disease (AD) patients (Chang et al., 2002), Parkinson’s disease, Huntington’s disease (Peel et al., 2001; Peel and Bredesen, 2003), dementia (Taga et al., 2017), and prion disease (Paquet et al., 2009). Inhibiting PKR has proven to be effective in rescuing synaptic and learning deficits in two different AD mouse models (Hwang et al., 2017). In the context of these neurodegenerative diseases, it will be essential to investigate PACT’s involvement in activating PKR. Currently PACT-mediated PKR activation has been reported only in the case of Alzheimer’s patient brains and mouse models (Paquet et al., 2012). Activated PKR could also contribute to the behavioral and neurophysiological abnormalities in Down syndrome as PKR inhibitory drugs were able to partially rescue the synaptic plasticity and long-term memory deficits in a mouse model (Zhu et al., 2019). Thus, our results presented here possibly have broader implications beyond DYT-*PRKRA.* Luteolin may also be useful for treating diseases triggered by inflammation where involvement of PACT-PKR pathway has been established such as in hepatic stellate cells, which are major contributors for the progression of hepatic fibrosis (Nakamura et al., 2015). Additionally, luteolin could also be effective against inflammatory conditions such as colitis in which the involvement of PACT-PKR pathway is established (Farabaugh et al., 2017; Farabaugh et al., 2020; Chukwurah et al., 2021). PKR has also been shown to be an important regulator of hematopoietic stem/progenitor cell fate and proliferation and is thought to play a role in bone marrow failure conditions including myelodysplastic syndrome (Liu et al., 2013). The involvement of PACT in hematopoietic lineages has not been investigated in depth and it could be interesting area for future investigation to evaluate if luteolin affects hematopoietic stem/progenitor cell fate. Other flavonoids such as quercetin are also known to reduce ISR and ATF4 expression in Alzheimer’s mouse models and improve memory (Nakagawa and Ohta, 2019). In our previous study with flavonoids, quercetin showed ability to disrupt PACT-PKR interaction and to inhibit PKR activation under conditions of oxidative stress and inflammation (Dabo et al., 2017). Our research thus opens a new area of investigation to evaluate the suitability of luteolin and other flavonoids in treating DYT-*PRKRA* and possibly other neurodegenerative and inflammatory conditions.

In the context of DYT-*PRKRA*, the patient cells exhibit enhanced interactions between mutant PACT and PKR even in the absence of ER stress. Consequently, the levels of p-PKR are about 5-fold higher (Figure 1) in patient cells in the absence of ER stress. Moreover, the patient cells from compound heterozygous individual carrying P222L and C213R mutations used in this study as well as previously used P222L homozygous patient cells exhibit higher level of apoptosis in the absence of cellular stress. Thus, neurodegeneration in DYT-*PRKRA* patients can be expected as a long-term outcome of the increased level of apoptosis in the absence of cellular stress. A limited number of imaging studies for the compound heterozygous patient carrying P222L and C213R mutant alleles used in the current study have indications of some neuronal apoptosis. Brain imaging performed at different ages indicated progressive MRI abnormalities with significant bilateral volume loss in the basal ganglia (Brashear, 2013; Lemmon et al., 2013), which could have resulted from enhanced apoptosis. This individual also developed dystonia after a febrile illness, which could have been a possible cellular stress event triggering hyperactivation of PACT-PKR pathway and progressive neuronal dysfunction or loss. Additionally, in accordance with our earlier *in vitro* studies with lymphoblasts from three Brazilian P222L homozygous patients that showed enhanced apoptosis (Vaughn et al., 2015), the imaging studies on one Portuguese P222L homozygous patient showed significant bilateral loss of striatal presynaptic dopamine transporters, suggesting nigrostriatal neurodegeneration (Pinto et al., 2020). Recently, Masnada et al. (2021) also reported bilateral striatal degeneration in two non-related DYT-PRKRA patients with two compound heterozygous patients. One of these patients had P222L and G43S mutations and presented dystonia at 30 months and the other had C213F and V72F mutations and presented at 14 months of life. Both patients showed recurrent fever-induced episodes of acute encephalopathy resulting in cognitive impairment, and generalized dystonia, among other symptoms. Evidence of cerebellar atrophy was also documented in one of these patients. A DYT-PRKRA patient homozygous for G43C mutation also showed MRI abnormalities with mild cerebral atrophy (Bhowmick et al., 2022). Additionally, there is evidence of neuronal apoptosis in *lear-5J* mice which carry a spontaneously arisen *PRKRA* frameshift mutation that truncates PACT protein. Homozygous *lear-5J* mice exhibit progressive dystonia, kinked tails, and mortality and apoptosis in the dorsal root ganglia and the trigeminal ganglion (Palmer et al., 2016).

As the PACT-PKR stress response pathway functions similarly in all cell types including neuronal cells (Chen et al., 2006; Paquet et al., 2012; Vaughn et al., 2014) and PACT mediated PKR activation and its involvement in neurodegeneration has been noted in Alzheimer’s patients and mouse models (Paquet et al., 2012), it is important to study the ISR dysregulation in DYT-*PRKRA* neurons. Currently no DYT-*PRKRA* neurons are available and our studies on patient lymphoblasts indicate that considerable efforts involved in undertaking in-depth studies using DYT-*PRKRA* patient-derived neurons from induced pluripotent stem cells (iPSCs) would be worthwhile in future. Our results thus open a new area of investigation to evaluate the suitability of luteolin in treating DYT-*PRKRA* and possibly other neurodegenerative conditions. The *lear-5J* mouse model (Palmer et al., 2016) of DYT-*PRKRA* will be very useful for characterizing the contribution of ISR dysregulation to dystonia phenotype, evaluating luteolin as a therapeutic agent, and determining therapeutic windows in which luteolin mediated ISR modulation could prove beneficial.

## Data Availability

The raw data supporting the conclusion of this article will be made available by the authors, without undue reservation.

## Abbreviations

PKR, Protein kinase RNA-activated; IFN, interferon; ds, double-stranded; eIF2, eukaryotic initiation factor; ER, endoplasmic reticulum; dsRBM, dsRNA-binding motif; PACT, PKR activator protein; PRKRA, Protein Activator of interferon-induced protein kinase; PERK, PKR-like endoplasmic reticulum resident kinase; GADD34, Growth arrest and DNA damage-inducible protein 34; DYT, dystonia; ISR, integrated stress response.
